# Efficacy, immunogenicity, and safety of pneumococcal conjugate vaccine in children: a systematic review and meta-analysis

**DOI:** 10.3389/fped.2025.1652946

**Published:** 2025-10-01

**Authors:** Qin Dai, Yujie Dong, Jing Wu, Qiyan Peng

**Affiliations:** ^1^ School of Nursing and Health Care, Leshan Vocational and Technical College, Leshan, Sichuan, China; ^2^ School of Medicine, Leshan Vocational and Technical College, Leshan, Sichuan, China

**Keywords:** pneumococcal conjugate vaccine, immunogenicity, pneumonia prevention, meta-analysis, pediatric vaccination

## Abstract

**Purpose:**

Pneumococcal infections are the leading cause of childhood morbidity and mortality, and efforts have been made to search for effective means of prevention and control to reduce their serious threat to children's health. This study intends to investigate the efficacy, immunogenicity, and safety of pneumococcal conjugate vaccine (PCV) in children.

**Methods:**

A search was conducted in PubMed, Web of Science, Cochrane Library, and Embase up to June 19, 2024. Children aged 0–2 years were included, and PCV was given in the intervention group and a placebo in the control group. The outcomes were immunogenicity, safety, and adverse events. Meta-analyses were conducted using RevMan and Stata17, and the Cochrane Risk of Bias 2.0 was utilized for quality assessment.

**Results:**

Eleven studies involving 147,274 participants were included. Meta-analyses revealed that compared with placebo, PCV greatly lowered the incidence of pneumonia (RR 0.78, 95% CI 0.70, 0.87; *P* < 0.001). PCV also significantly raised the levels of IgG antibody 6B (GMR 22.16, 95% CI 3.73, 131.47; *P* < 0.001), 9V (GMR 15.18, 95% CI 1.48, 155.27; *P* = 0.02), 14 (GMR 12.50, 95% CI 1.76, 88.98; *P* = 0.01), 18C (GMR 20.20, 95% CI 1.47, 276.72; *P* = 0.04), 19F (GMR 15.43, 95% CI 1.14, 209.15; *P* = 0.04), and 23F (GMR 13.74, 95% CI 2.42, 78.01; *P* = 0.003). However, PCV produced no statistically significant increase in the levels of IgG antibody 1/4/5.

**Conclusion:**

PCV reduces the incidence of pneumonia and improves the levels of IgG antibodies. However, given the lack of data on adverse events in the included studies, we hope that standardized reporting methods for safety outcomes can be adopted in future randomized controlled trials to improve data comparability and utility and provide a more solid basis for evidence-based decision-making.

**Systematic Review Registration:**

https://www.crd.york.ac.uk/PROSPERO/view/CRD42024570854, PROSPERO CRD42024570854.

## 1 Introduction

Pneumococcal infections represent the leading cause of childhood morbidity and mortality worldwide. According to the World Health Organization (WHO) epidemiological data, pneumococcal disease claims the lives of up to one million children under five years of age annually, with the heaviest burden falling upon developing countries (1). The clinical spectrum of pneumococcal disease is severe and multifaceted, encompassing otitis media, pneumonia, meningitis, and other invasive diseases (2). Pneumococcal meningitis is particularly devastating, carrying a mortality rate approaching 50%, with survivors frequently experiencing severe neurological deficits (3, 4).

The pneumococcal conjugate vaccine (PCV) stands as a critical intervention against this public health challenge, offering long-term protection for children. Its mechanism involves inducing B and T cell immunity to generate effective immune responses and durable immune memory, enabling the immune system to rapidly recognize Streptococcus pneumoniae and mount protective antibody responses (5). Despite this established immunological foundation, the real-world performance of PCV across diverse pediatric populations remains an area in need of comprehensive synthesis. Notable variations have been found in research: PCV9 demonstrates 83% efficacy against invasive pneumococcal disease (IPD) in HIV-uninfected children (6) but only 31% efficacy against pneumonia linked to seven respiratory viruses in hospitalized children (7). Furthermore, clinical trials have revealed significant serotype-specific differences in the PCV protection, with PCV showing consistent efficacy against serotype 6A but inconsistent efficacy against serotype 19A (8, 9). These discrepancies possibly stem from factors including vaccine valency, geographical variations in circulating serotypes, study design heterogeneity, and sample size limitations.

Given this complex landscape, it is needed to synthesize fragmented evidence on PCV performance. Therefore, this study aims to synthesize total available data on the effect of PCV on children. We will conduct a systematic assessment from three critical dimensions: protective efficacy or effectiveness against IPD, pneumonia, and otitis media; immunogenicity profiles, evaluating the magnitude and durability of immune responses across vaccine serotypes; and comprehensive safety parameters, documenting adverse event profiles. By rigorously evaluating the efficacy, immunogenicity, and safety of PCV, this study seeks to generate robust, consolidated evidence. Our ultimate goal is to provide a stronger, more reliable foundation for evidence-based prevention strategies for pneumococcal disease, thereby contributing to the reduction of the global burden of childhood pneumococcal disease.

## 2 Methods

This study adhered to the PRISMA statement (9) (Supplementary Table S1), and the study protocol was registered with PROSPERO (CRD42024570854).

### 2.1 Search strategy

We searched PubMed, Web of Science, Cochrane Library, and Embase up to June 19, 2024, and only English-language studies were included, using medical subject headings (Child, Infant, Newborn, Adolescent, Vaccine, Pneumococcal Vaccines) plus free terms (Supplementary Table S2). The reference lists of systematic reviews were further searched for potentially missing studies.

### 2.2 Eligibility criteria

Inclusion criteria: (1) Participant: children aged 0–2 years. (2) Intervention: PCV; Comparison: placebo. (3) Outcome: incidence of pneumonia (primary outcome), and immunogenicity and safety (secondary outcomes). (4) Study design: randomized controlled trials (RCTs).

Exclusion criteria: (1) case reports, animal or cell experiments, scientific experiment plans, letters, reviews, editorials, and conference papers; (2) missing data or serious errors; (3) duplicate publications; (4) unavailable full text.

### 2.3 Study screening and data extraction

The retrieved studies were imported into EndNote, and two reviewers (QD and YJD) independently read the title and abstract first based on the eligibility criteria, and then further examined the full text. Any discrepancy was settled by discussion or consultation with a third reviewer. The following data were independently extracted by two reviewers (QD and JW) using Excel 2016: first author, country, year of publication, sample size, age, sex, disease, vaccine, vaccine type, outcome metrics (pneumonia, IgG antibody 1/4/5/6B/9V/14/18C/19F/23F).

### 2.4 Quality assessment

We utilized Cochrane Risk of Bias 2.0 (10) for quality assessment of RCTs from randomization, deviations from intended interventions, measurement of the outcome, missing outcome data, and selection of the reported result. Each domain was assessed as low risk, uncertain, or high risk.

### 2.5 Statistical analysis

The outcome pneumonia was pooled by risk ratio (RR) with 95% CI, and the immunogenicity by geometric mean ratio (GMR) with 95% CI as geometric means with 95% CI were reported in the original study. We assessed the heterogeneity of included studies by I^2^ statistic, and then adopted a random- or fixed-effects model when the heterogeneity was present (I^2^ > 50% or *P* < 0.1) or absent. Leave-one-out sensitivity analyses were conducted (11). We evaluated publication bias by funnel plot and Egger's test when over 10 studies were included. *P* < 0.05 was deemed statistical significance. Stata 15.1 was utilized for meta-analyses (12).

## 3 Results

### 3.1 Search results

**Figure 1:**
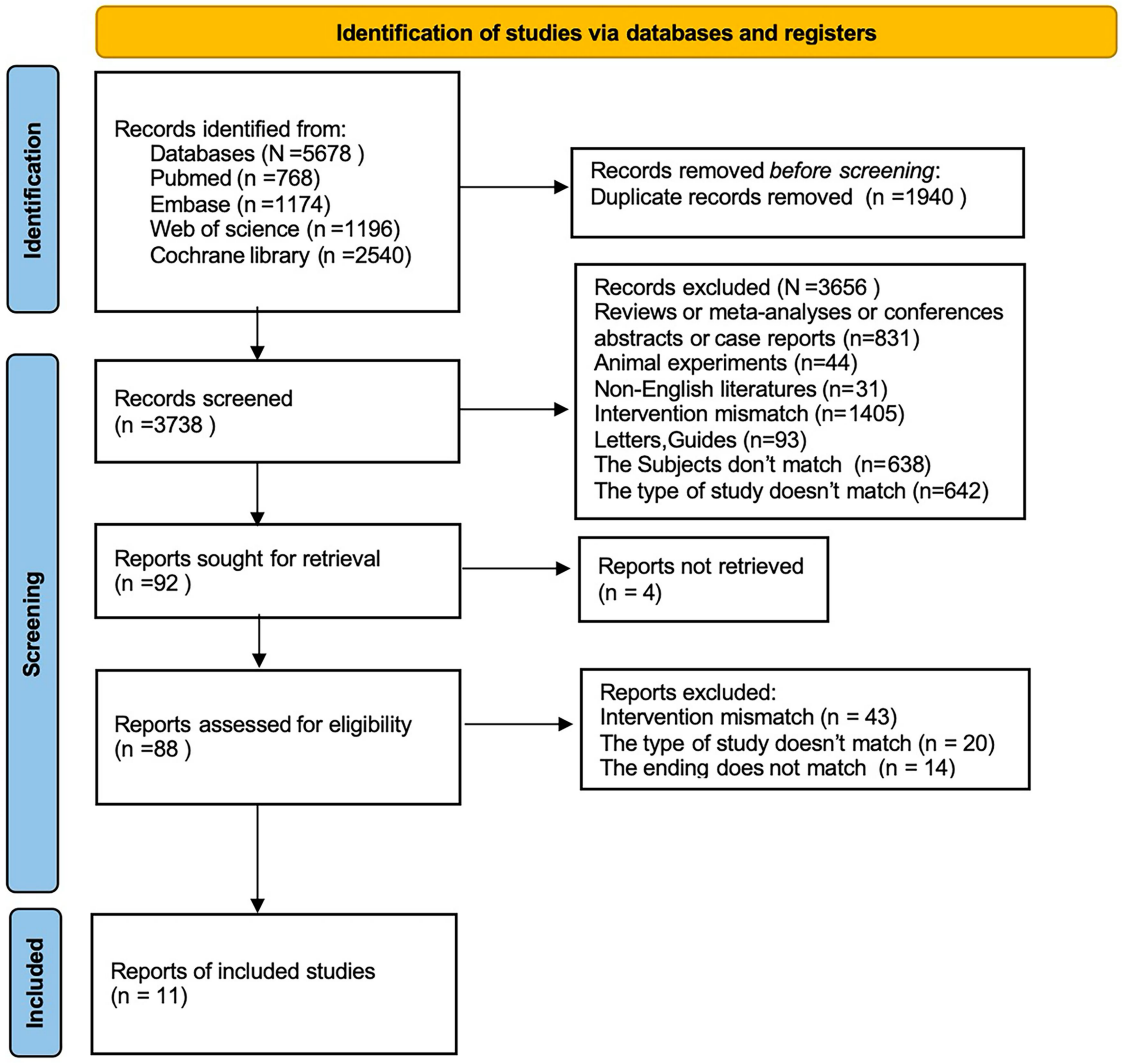
Flowchart.

We initially retrieved 5,678 studies, of which 1,940 were excluded as duplicates. 1,802 studies were excluded after title and abstract review. Then the full text of the remainder was examined according to the eligibility criteria. Finally, 11 studies (2, 5, 6, 13–20) were included (Figure 1).

### 3.2 Study characteristics

All the included studies were published from 1998 to 2023, and involved 147,274 participants aged 0–2 years (73,676 in the PCV group and 73,598 in the control group). The participants were from five countries (South Africa, Gambia, the Philippines, China, and Australia) (Table 1).

**Table 1:** Study characteristics.

First author	Year of publication	Country	Sample size		Sex (m/f)		Age		Vaccine dosage
			t	c	t	c	t	c	
Klugman et al. (5)	2003	South Africa	19,922	19,914	10,021/9,901	9,937/9,977	–	–	3 + 0
Madhi et al. (6)	2004	South Africa	18,245	18,268	–	–	–	–	3 + 0
Nunes et al. (13)	2021	South Africa	1,289	1,288			6, 10, and 14 weeks	6, 10, and 14 weeks	3 + 0
			18,633	18,626			6, 10, and 14 weeks	6, 10, and 14 weeks	3 + 0
Akinsola et al. (14)	2012	South Africa	138	144			38–52 months	38–52 months	2 + 1
Saaka et al. (15)	2008	Gambia	103	93	No sex difference		24 or 18 weeks	24 weeks or 18 weeks	3 + 0
Mbelle et al. (16)	1999	South Africa	242	239	No sex difference		45, 76, and 96 days (age was not statistically significant)	45, 76, and 96 days	3 + 0
Huebner et al. (17)	2002	South Africa	250	250	No sex difference		6, 10, and 14 weeks	6, 10, and 14 weeks	3 + 0
Soininen et al. (18)	2009	Philippines	555	556	243/236	2,476/244	Dose 1: 6 weeks Dose 2: 10 weeks Dose 3: 14 weeks	Dose 1: 6 weeks Dose 2: 10 weeks Dose 3: 14 weeks	2 + 1
Cutts et al. (19)	2005	Gambia	8,189	8,151	4,100/4,089	4,074/4,077	Dose 1: 75 (59–108) days Dose 2: 122 (97–166) days Dose 3: 169 (136–225) days	Dose 1: 75 (59–108) days Dose 2: 122 (97–166) days Dose 3: 169 (136–225) days	3 + 0
Lucero et al. (20)	2009	Philippines	6,013	6,018	No sex difference		Dose 1: 1.8 months Dose 2: 2.9 months Dose 3: 3.9 months	Dose 1: 1.8 months Dose 2: 2.9 months Dose 3: 3.9 months	2 + 1
Mackenzie et al. (2)	2009	Australia	97	51	45/52	30/21	10.9 (1–22)	9.5 (3–17)	3 + 0

### 3.3 Quality assessment

**Figure 2:**
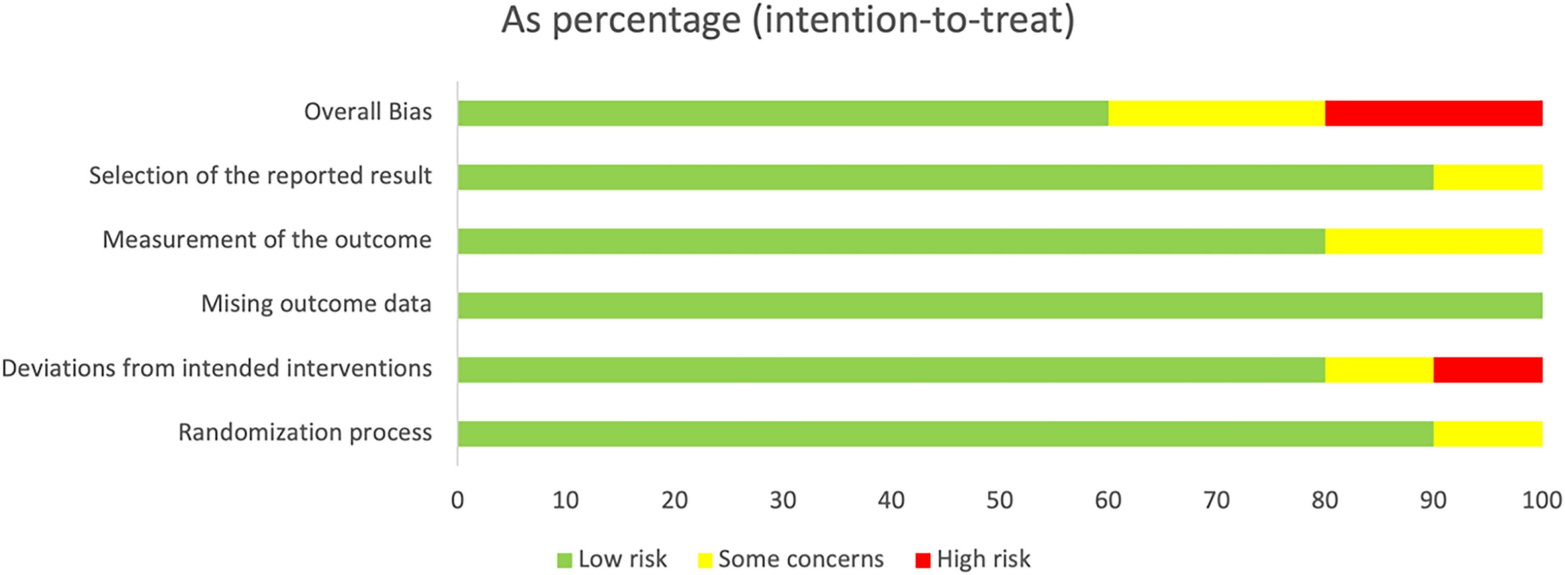
Quality assessment.

For randomization, the study by Nunes et al. mentioned allocation concealment, but the specific methods were unclear, so it was assessed as a clear risk of bias in the randomization process; the studies by Klugman et al. (5) and Madhi et al. (6) adopted allocation concealment, but nursing personnel were aware of the grouping details, so a potential risk of bias was present. For deviations from intended interventions, patients infected with HIV underwent other treatments, resulting in a high risk of bias in the study by Nunes et al. (13). For missing outcome data, none of the studies mentioned any missing data, so the risk of bias was low. For measurement of the outcome, in the studies by Nunes et al. (13), Lucero et al. (20), Klugman et al. (5), and Madhi et al. (6), measurement personnel were aware of the interventions received by participants, which, however, did not influence the measurement of outcomes, so potential measurement bias still existed (5, 6, 13, 20). For selection of the reported result, one study by Madhi et al. (6) reported only the treatment efficacy on pneumonia and did not mention other side effects, so selection bias may be present. To sum up, the overall bias of all the included studies was low (Figure 2). The aforementioned issues still require careful consideration. It is recommended that standardized reporting methods for safety outcomes be adopted in future RCTs to improve data comparability and support evidence-based decision-making.

### 3.4 Meta-analysis results

#### 3.4.1 Pneumonia

**Figure 3:**
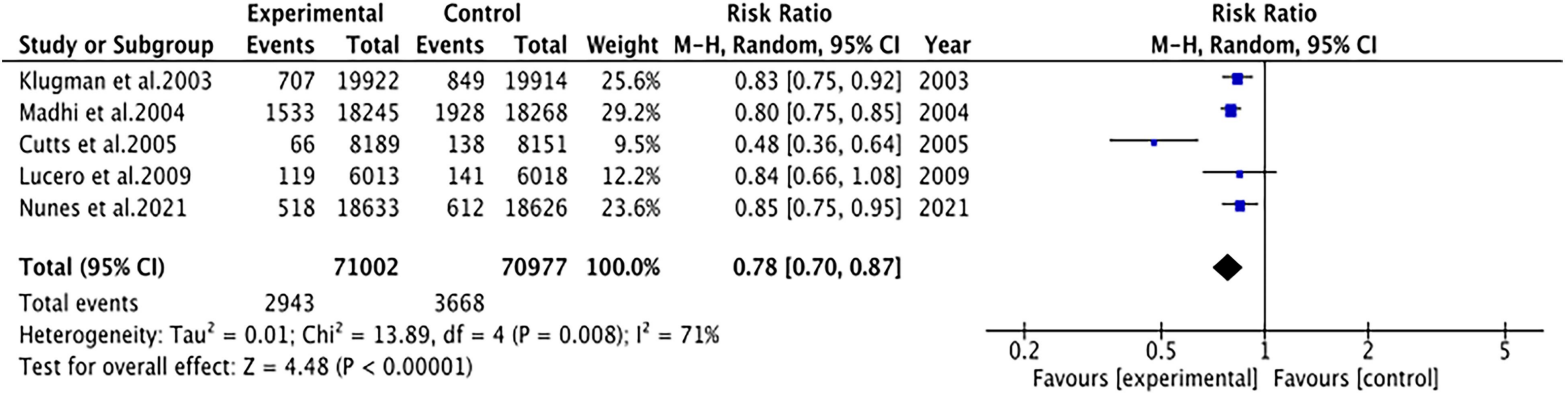
Forest plot for pneumonia.

**Figure 4:**
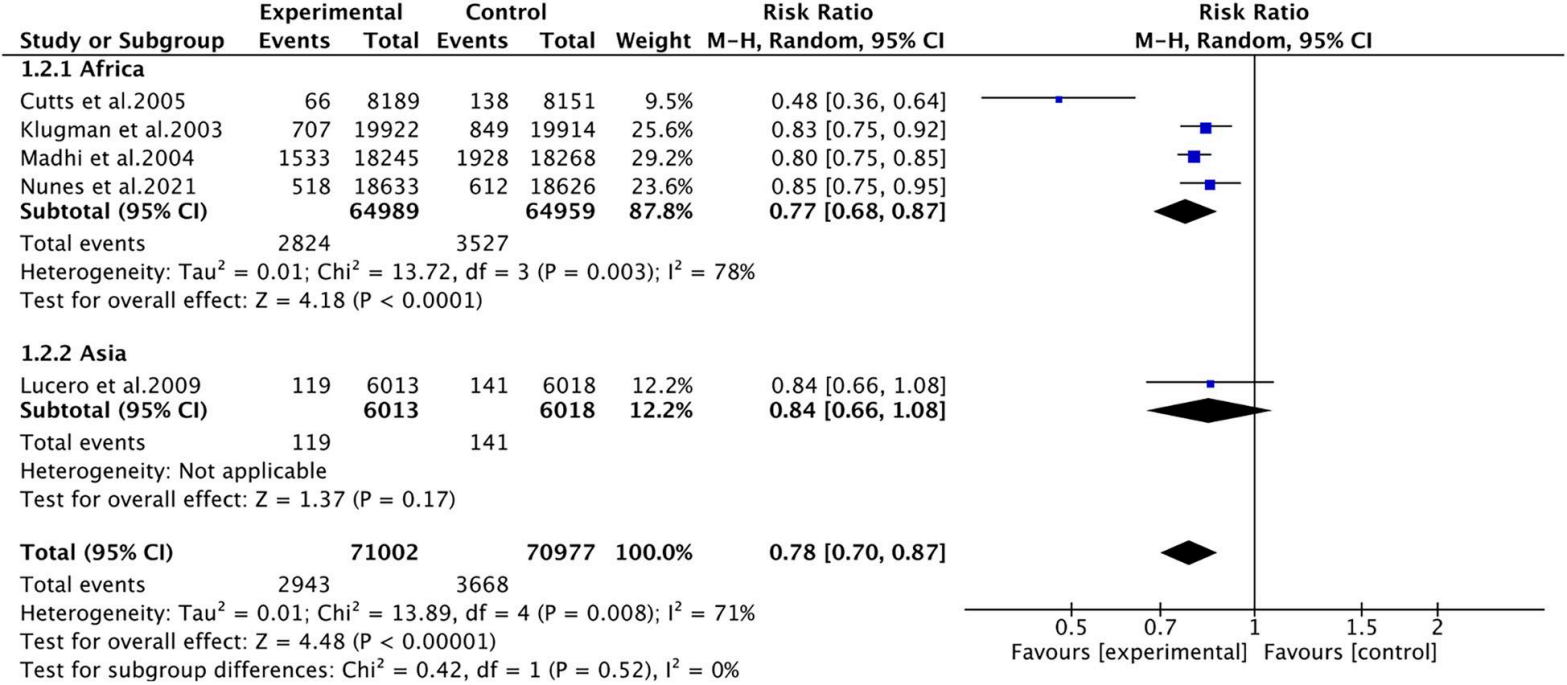
Subgroup analysis on pneumonia (region).

**Figure 5:**
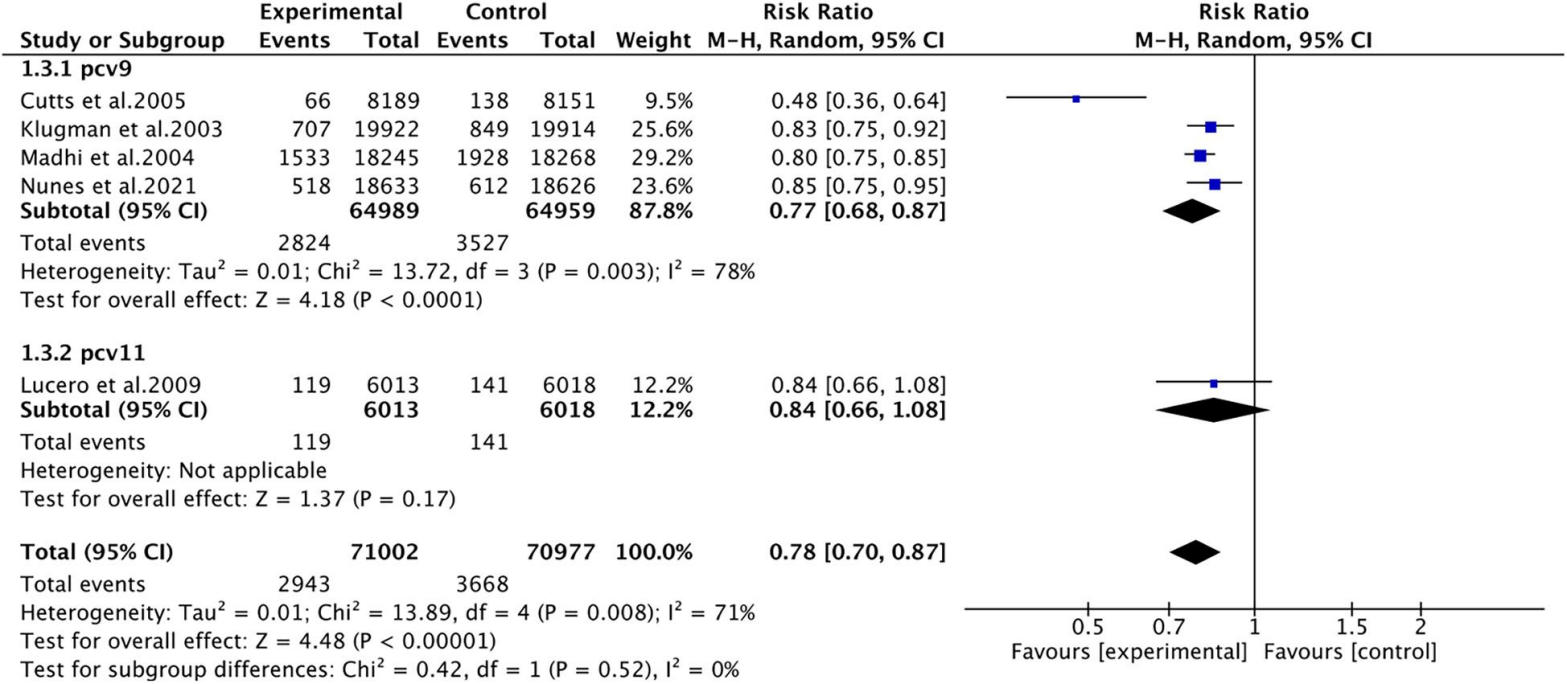
Subgroup analysis on pneumonia (vaccine type).

Pneumonia was reported in five studies involving 141,979 patients. We adopted a random-effects model (I^2^ = 71%, *P* = 0.008). The pooled results revealed that the incidence of pneumonia was far lower in the PCV group (RR 0.78, 95% CI 0.70–0.87, *P* < 0.001) (Figure 3). Due to significant heterogeneity, subgroup analyses were conducted. The results of subgroup analyses by region showed that PCV significantly reduced the incidence of pneumonia in Africa (RR 0.77, 95% CI 0.68, 0.87, *P* < 0.001), while the effect of PCV on pneumonia incidence had no significant difference from placebo in Asia (RR 0.84, 95% CI 0.66, 1.08, *P* = 0.17) (Figure 4). The results of subgroup analyses by vaccine type showed that PCV9 significantly reduced the incidence of pneumonia (RR 0.77, 95% CI 0.68, 0.87, *P* < 0.001), while the effect of PCV11 on pneumonia incidence had no significant difference from placebo (RR 0.84, 95% CI 0.66, 1.08, *P* = 0.17) (Figure 5). However, the results should be interpreted with caution since only one study from Asia was included.

#### 3.4.2 Immunogenicity

##### 3.4.2.1 IgG antibody 1

**Figure 6:**
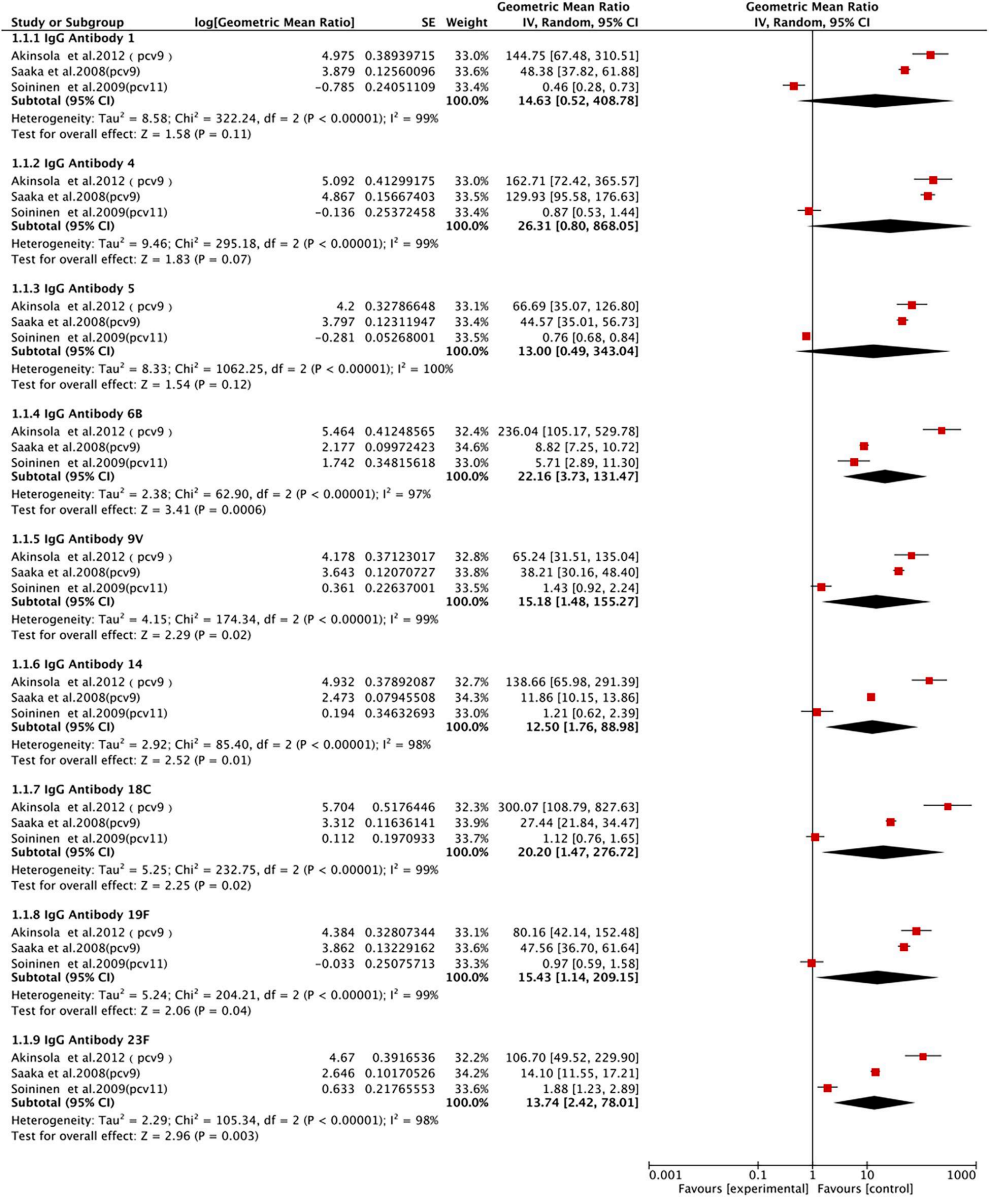
Forest plot for serum antibodies.

IgG antibody 1 was described in three studies involving 1,436 patients. We adopted a random-effects model (I^2^ = 99%, *P* < 0.00001). We found that the PCV group had a higher level of IgG antibody 1, without a statistically significant difference (GMR 14.63, 95% CI 0.52, 408.78; *P* = 0.11) (Figure 6).

##### 3.4.2.2 IgG antibody 4

IgG antibody 4 was described in three studies involving 1,433 patients. We adopted a random-effects model (I^2^ = 99%). We found that the level of IgG antibody 4 was significantly elevated in the PCV group, without a statistically significant difference (GMR 26.31, 95% CI 0.80, 868.05; *P* = 0.07) (Figure 6).

##### 3.4.2.3 IgG antibody 5

IgG antibody 5 was described in three studies involving 1,431 patients. We adopted a random-effects model (I^2^ = 100%, *P* < 0.00001). We found that the level of IgG antibody 5 was significantly elevated in the PCV group, with no statistically significant difference (GMR 13, 95% CI 0.49, 343.04; *P* = 0.12) (Figure 6).

##### 3.4.2.4 IgG antibody 6B

Three studies with 1,445 patients reported IgG antibody 6B. A random-effects model was utilized (I^2^ = 97%, *P* < 0.00001). We observed that the PCV group had a significantly elevated level of IgG antibody 6B (GMR 22.16, 95% CI 3.73, 131.47; *P* < 0.001) (Figure 6).

##### 3.4.2.5 IgG antibody 9V

Three studies with 1,425 patients reported IgG antibody 9V. A random-effects model was utilized (I^2^ = 99%, *P* < 0.00001). We observed that the PCV group had a significantly elevated level of IgG antibody 9V (GMR 15.18, 95% CI 1.48, 155.27; *P* = 0.02) (Figure 6).

##### 3.4.2.6 IgG antibody 14

Three studies with 1,421 patients reported IgG antibody 14. A random-effects model was utilized (I^2^ = 98%, *P* < 0.00001). We observed that the PCV group had a significantly elevated level of IgG antibody 14 (GMR 12.50, 95% CI 1.76, 88.98; *P* = 0.01) (Figure 6).

##### 3.4.2.7 Igg antibody 18C

Three studies with 1,431 patients reported IgG antibody 18C. A random-effects model was utilized (I^2^ = 91%, *P* < 0.00001). The results showed a significantly elevated level of IgG antibody 18C in the PCV group (GMR 20.20, 95% CI 1.47, 276.72; *P* = 0.01) (Figure 6).

##### 3.4.2.8 IgG antibody 19F

IgG antibody 19F was described in three studies with 1,436 patients. We adopted a random-effects model (I^2^ = 98%, *P* < 0.00001). The results showed a significantly elevated level of IgG antibody 19F in the PCV group (GMR 15.43, 95% CI 1.14, 209.15; *P* = 0.04) (Figure 6).

##### 3.4.2.9 IgG antibody 23F

IgG antibody 23F was described in three studies with 1,438 patients. We adopted a random-effects model (I^2^ = 95%, *P* < 0.00001). The pooled results showed a significantly elevated level of IgG antibody 23F in the PCV group (GMR 13.74, 95% CI 2.42, 78.01; *P* = 0.003) (Figure 6).

### 3.5 Safety

Eleven studies were included, most of which did not explicitly clarify the side effects and adverse reactions of PCV. Only one study (Nontombi Mbelle) described that mortality, local reactions, and systemic side effects had no statistically significant differences in the PCV and control groups except that the control group had poor food intake at the time of immunization (4% vs. 1.2%) (16); 14 h after immunization, two placebo-treated children and one vaccinated child reported transient urticaria (*P* = 0.05) (16). Keith P. Klugman mentioned an increased incidence of asthma following immunization with PCV (2.96 per 1,000 children vs. 1.66 in controls), which was seen in the case of a decline in the risk of radiologically-confirmed pneumonia in vaccinated children (17.9 per 1,000 children vs. 21.5 in controls). It can be seen that the risk of asthma in PCV recipients remains to be further determined. The safety analysis involved over 1,000 comparisons, and significant differences at the 5% level were generated by hypothesis but might be due to chance (5). F T Cutts clearly described the good safety profile of PCV, contrary to the findings in South Africa that the risk of asthma rises in PCV recipients (19). To sum up, the safety of PCV was verified (Table 2).

**Table 2:** The incidence of adverse events.

Study	Incidence of asthma	Mortality	Urticaria
Mbelle et al. (16)		Unclear	1/242 (0.4%)
Klugman et al. (5)	2.96/1,000 (0.3%)		

### 3.6 Sensitivity analysis and publication bias

**Figure 7:**
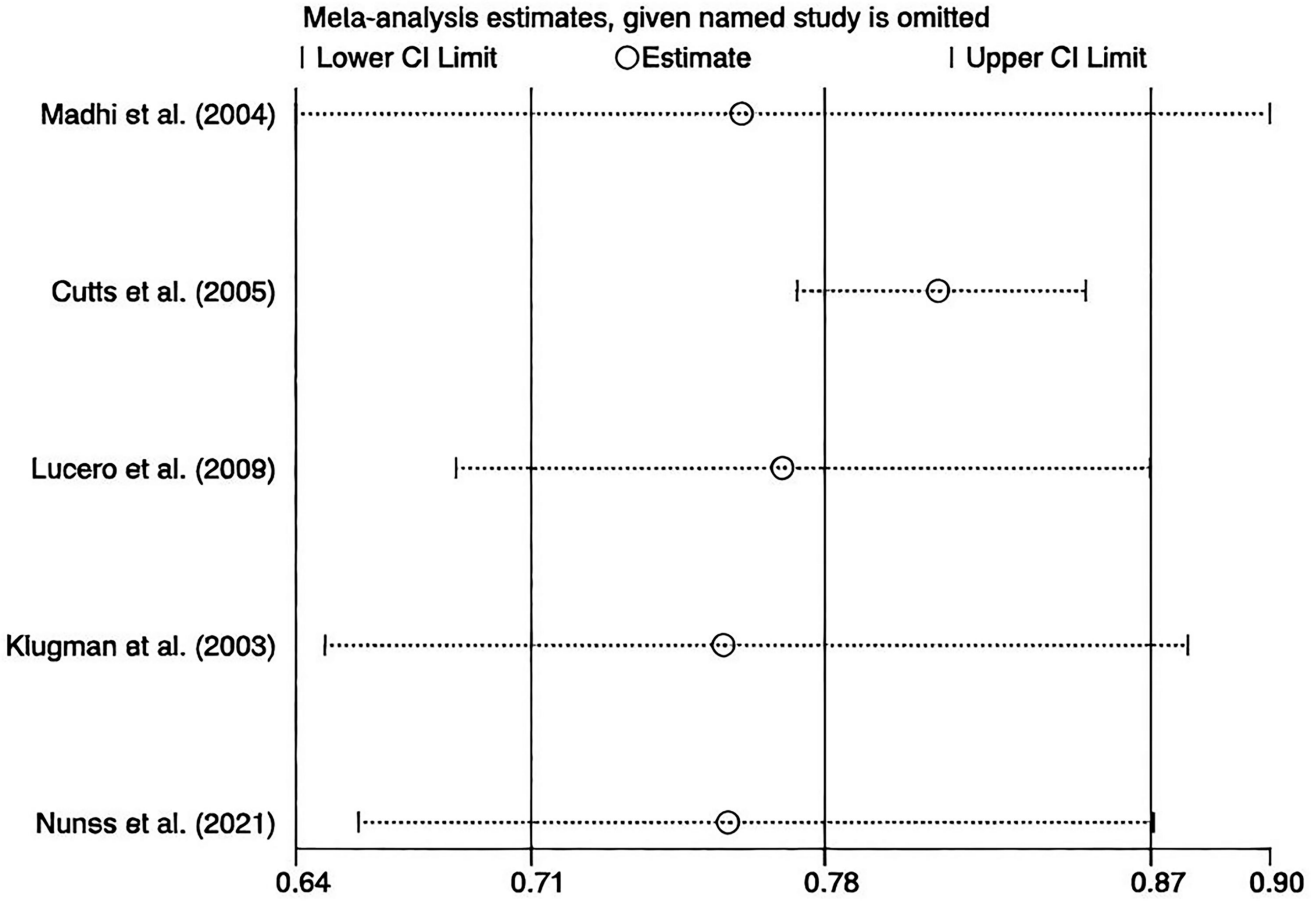
Sensitivity analysis.

We performed leave-one-out sensitivity analyses, and the results stayed robust (Figure 7). No publication bias was assessed since fewer than 10 studies were included.

## 4 Discussion

Pneumococcal infections are the leading cause of childhood morbidity and mortality, especially in developing countries (21). PCV, as an important component of the global childhood immunization program, has been widely recognized for its efficacy in preventing IPD in children. This study synthesized the available clinical data in 11 studies to fully assess the efficacy, safety, and immunogenicity of PCV in children. The results showed that PCV displayed significant protective effects against serotype-specific pneumonia, with good safety and immunogenicity profiles. By comparing the immune responses including antibody levels and persistence of PCV, the performance of PCV in practical use was further revealed. These findings are important for understanding the long-term protection of vaccines, optimizing vaccination strategies, and developing future vaccination policies. We expect that this study will offer a scientific basis for public health decision-makers and clinical practice, and more comprehensive protection for children.

This study verified that PCV significantly lowered the risk of pneumococcal disease in children, consistent with the findings of Ricketson et al. (22). PCV can induce T lymphocyte-dependent immune responses to trigger the immune memory and antibody response, which can induce the production of antibodies against pneumococcus and enhance immune defenses against pneumococcus (23). Bruce et al. (24) also found the good safety and immunogenicity of 7-valent PCV (PCV7) in children, which lays an important basis for future development and application of vaccines. PCV achieves direct protection of vaccinated children and herd immunity by decreasing the number of virus carriers and the spread in the community (25), suggesting that even unvaccinated children can indirectly benefit from herd immunity, thereby reducing the overall incidence of pneumococcal disease. After being introduced into the immunization program globally, PCV13 has been proven effective in preventing pneumococcal diseases including IPD and hospitalization for all-cause pneumonia in both adults and infants (26, 27). As the valence number increases, PCV (PCV7 to PCV13) covers a wider range of pneumococcal serotypes, achieving more comprehensive protection. In this way, illness due to non-vaccine serotypes of pneumococcus can be reduced, lowering the overall incidence of pneumonia in children. A systematic review of surveillance data from different countries revealed a rapid and substantial decline in IPD in all regions after PCV was introduced (28). In addition, PCV has yielded a great decrease in pneumococcal nasopharyngeal carriage of vaccine serotypes in children, directly affecting pneumococcal transmission and infection (29). By reducing pneumococcal carriage, the vaccine restrains the community spread of disease, further reducing the incidence of pneumonia. Finally, both morbidity and mortality of IPD are greatly reduced by PCV, particularly in children aged below 5 years (30), which reduces not only pneumonia due to pneumococcus but also other serious IPDs (e.g., meningitis and sepsis).

The morbidity and mortality of pneumococcal disease in children have become a major health concern globally. However, available data demonstrate that the children's disease burden and vaccine effectiveness greatly vary across regions and age groups. These discrepancies may be attributed to vaccination coverage, disease surveillance capacity, serotype distribution, and individual physiologic and immune status.

The effectiveness of vaccines in controlling pneumococcal disease varies across regions due to different vaccination coverage. For example, although the PCV13 vaccination coverage has increased in 2017–2021 in China, a significant difference is present between economically developed and less developed regions (31), indicating that unequal distribution of vaccines may undermine the effectiveness of herd immunity nationwide. In addition, low vaccination coverage in less developed countries contributes to the high burden of pneumococcal disease, whereas high-income countries have significantly reduced the disease burden by high-coverage vaccination strategies (32). The differences in prevalent serotypes across regions are also a key influencing factor for vaccine efficacy. PCV13 covers over 20 serotypes, but specific serotypes are not included in the vaccine program in some regions, weakening its protective effect (33). For example, the protective efficacy of PCV13 is restricted in sub-Saharan Africa with a high prevalence of serotypes 1 and 5, and PCV20 may be more suitable for this region (34). Besides, discrepancies in disease surveillance capacity also affect the assessment of vaccine efficacy, i.e., high-income countries often possess better surveillance systems, whereas inadequate surveillance in resource-limited regions may result in underestimation of vaccine efficacy or underreporting of morbidity (35).

The role of age in vaccine efficacy should not be neglected. The immune system of infants and young children is not yet fully developed, so the post-vaccination level of antibody response may be lower than in older children. According to a large-scale clinical trial, the antibody levels in children aged below two years significantly increase after PCV13 vaccination, but its persistence is shorter than in children aged above two years (36), which may be related to the less developed humoral immune function of infants and children. Besides, multiple doses and booster immunizations may be required for weaker immunogenic groups (e.g., preterm infants) to enhance protection (37). The optimal vaccination schedule for different age groups also varies by country and region. Many high-income countries adopt a “2 + 1” model (2 basic doses+1 booster), while a “3 + 0” model is adopted in some low-income countries (3 basic doses, no booster) (38). Studies have documented the protective efficacy of the “2 + 1” model in children >2 years old and the “3 + 0” model in infants and young children, the latter of which may require subsequent boosters to prolong the protective effect (39).

Underlying health status is also an important influencing factor for vaccine efficacy. Beyond efficacy and safety, PCV demonstrates significant cost-effectiveness in low-resource settings where the burden of pneumococcal disease is highest. Studies from sub-Saharan Africa and South Asia estimate that PCV introduction reduces healthcare costs by 20%–40% through decreased hospitalizations for pneumonia and meningitis (27, 30). For example, in Gambia, immunization with PCV9 averted treatment costs of approximately $2.8 million annually (19). The herd immunity effects of PCV further amplify cost savings by reducing transmission to unvaccinated populations. Despite higher upfront costs, PCV programs in low- and middle-income countries (LMICs) yield a favorable cost-per-DALY averted, often falling below WHO-recommended thresholds for cost-effective interventions.

Immunocompromised children or those with underlying diseases have a weaker immune response to vaccines and thus may require additional immunization. Studies have revealed that the antibody levels in immunodeficient children immunized with standard doses of PCV are still lower than in healthy ones, suggesting that the former may require higher doses or booster immunizations (40). In addition, PCV is usually more effective in healthy children because antibody responses can be produced more efficiently in these children (41). Natural immunity may have been established in children with a history of pneumococcal exposure, which has an impact on the vaccine efficacy. Research suggests that antibody levels increase slightly after PCV immunization in previously infected individuals, suggesting that natural immunity may interfere with the immune response triggered by vaccines (42). In addition, a combination of PCV with other vaccines (e.g., DPT) may also affect immunogenicity. To sum up, it is necessary to take into account all these factors in the vaccination strategy (43).

PCV exhibits significant effects in increasing the levels of IgG antibodies of specific serotypes. As can be seen from the forest plot, PCV significantly increased the levels of IgG antibody 6B/9V/14/18C/19F/23F, indicating that PCV elicits effective immune responses against these serotypes. The post-vaccination increase in IgG antibody 6B may be related to the type 6B capsular polysaccharide contained in PCV, which generates stronger antibody responses (Chen and Janssens et al.) (44, 45). Moreover, the increase in IgG antibody 9V/14/18C/19F/23F was also significant, indicating effective immune responses of PCV to these serotypes. Overall, despite high heterogeneity, our findings align with recent advancements in pneumococcal vaccine research; in particular, the immunogenicity patterns observed by Leuridan et al. in European cohorts corroborated the robust IgG responses to serotypes 6B/23F (GMR >20) observed in this study and also supported our findings regarding diminished responses in high-risk populations like preterm infants. An emerging study by Zhang et al. suggested nanoparticle-based PCV formulations may address the IgG response variability we documented, particularly for problematic serotypes like 3/19A. When comparing efficacy across settings, pneumonia reduction (78%) observed in LMICs parallels IPD reduction (79%) reported in high-income countries (22), demonstrating consistent clinical protection despite regional variations in serotype-specific efficacy. Furthermore, our conclusions on PCV safety are supported by both the WHO Global Advisory Committee (2023) reports on low systemic reaction rates and ongoing PCV20 trials (NCT05624398) showing similar reactogenicity profiles, reinforcing the favorable risk-benefit ratio of PCV across formulations and populations. Studies demonstrated the positive effect of PCV.

The heterogeneity in IgG responses across serotypes may be attributed to differences in the immunogenicity of capsular polysaccharides, vaccine formulation, and carrier protein effects. For example, serotypes 6B and 23F, which elicit robust IgG elevations, are known to form stable conjugates with CRM₁₉₇ carrier proteins, enhancing T cell-dependent immunity (44). In contrast, serotypes 1 and 5 may exhibit weaker immunogenicity due to structural properties or suboptimal conjugation (46). Additionally, the absence of serotype 1 in PCV7 formulations can explain its lower antibody levels observed in some cohorts (45). Host factors, such as age-related immune maturation, may further modulate these responses (36).

The choice of carrier protein (e.g., CRM₁₉₇ vs. tetanus toxoid/TT) may critically influence IgG magnitude and persistence. CRM₁₉₇, a non-toxic mutant of diphtheria toxin, enhances T cell-dependent responses via MHC class II presentation, particularly for polysaccharides like 6B and 23F (44). In contrast, TT-conjugated vaccines (e.g., some PCV10 formulations) may exhibit broader T-cell activation due to pre-existing immunity from tetanus vaccination (43). Notably, the larger molecular size of TT can improve antigen uptake by dendritic cells, potentially explaining higher IgG titers for certain serotypes (e.g., 14, 19F) in TT-based vaccines (37).

Emerging evidence suggests that carrier proteins may indirectly affect pneumococcal biofilm disruption. For example, TT-induced antibodies can bind to biofilm matrices, enhancing opsonophagocytosis (25). However, this remains underexplored in PCV trials and warrants future study.

However, PCV produced no statistically significant increase in the levels of IgG antibody 1/4/5, which may be attributed to the difference in vaccine formulations. PCV formulation may include different serotypes, which affect the immune response of the vaccine against pneumococcus of specific serotypes. If one serotype is not covered in the vaccine formulation, the immune response against this serotype may be weaker. Moreover, the immunogenicity of pneumococcal capsular polysaccharides may vary across serotypes, and capsular polysaccharides of some serotypes may be more easily recognized and responded to by the immune system (46). In addition, individual immune status, genetic background, and health conditions may influence the immune response of vaccines. For example, age at vaccination and timing may also affect the immune response, as the children's immune system develops differently at different ages. The dose and regimen of vaccination may also result in different immune effects (47).

In summary, PCV should be widely used in the global childhood immunization program, and monitoring and assessment of the vaccine efficacy is important, especially in regions with a high disease burden. We can guarantee by sustained research and evaluation that PCV provides optimal protection for all children and reduces the global burden of pneumococcal disease. In terms of safety, PCV is well tolerated and produces mild to moderate adverse reactions that are self-limiting (48). These findings are consistent with data from clinical trials and post-marketing vaccine surveillance, further demonstrating the safety of PCV in routine vaccination.

This study agrees with the WHO guidelines on the widespread use of PCV which has demonstrated significant efficacy in reducing the incidence and mortality of pneumococcal diseases in children, providing evidence for clinical popularization. The variability in serotype-specific IgG antibody levels should be closely monitored, and dynamic monitoring should be strengthened to help adjust vaccination strategies. From a policy perspective, PCV demonstrates significant cost-effectiveness, justifying investment in LMICs. Therefore, increased investment, optimized allocation, and improved coverage are needed to reduce the global disease burden.

## 5 Limitation

Despite the strict inclusion criteria and the application of a random-effects model to minimize the impact of heterogeneity due to differences in study design, geographical location, and participant characteristics, this study still has the following obvious limitations: First, the follow-up period of the included studies was generally short, which prevented the assessment of the long-term immunogenicity and delayed adverse events of the vaccine. This issue needs to be addressed as a priority in future research. Second, the current evidence is only derived from five countries, which restricts the generalizability of the study conclusions (especially their applicability to high-income countries). It is necessary to expand the geographical coverage to enhance the universality. To overcome these limitations, subsequent studies should prioritize long-term effect monitoring and incorporate data from a broader range of countries and regions to enhance the robustness and global applicability of the evidence base.

## 6 Conclusion

While PCV possesses a significant protective effect against vaccine-covered serotypes, our findings highlight the need to optimize vaccination strategies. Future research should prioritize long-term monitoring of non-vaccine serotypes (e.g., 3, 19A) to evaluate serotype replacement status, and immunogenicity testing of next-generation vaccines (e.g., PCV15, PCV20) against these emerging strains. The research results will be crucial for updating global vaccination policies. In addition, given the lack of data on adverse events in the included studies, we hope that standardized reporting methods for safety outcomes can be adopted in future RCTs.

PCV possesses a significant protective effect against IPD caused by vaccine-covered serotypes. In the future, the long-term immunogenicity of new-generation PCV, its protective effect against extended serotypes, and its impact on the epidemiologic trend of antimicrobial resistance should be further explored.

## Data Availability

The original contributions presented in the study are included in the article/Supplementary Material, further inquiries can be directed to the corresponding author.

## References

[1] World Health Organization. Pneumococcal conjugate vaccine for childhood immunization–WHO position paper. Wkly Epidemiol Rec. (2007) 82(12):93–104. 17380597

[2] Mackenzie GA Carapetis JR Leach AJ Morris PS. Pneumococcal vaccination and otitis media in Australian aboriginal infants: comparison of two birth cohorts before and after introduction of vaccination. BMC Pediatr. (2009) 9:14. 10.1186/1471-2431-9-14 19228431 PMC2656498

[3] Goetghebuer T West TE Wermenbol V Cadbury AL Milligan P Lloyd-Evans N Outcome of meningitis caused by Streptococcus pneumoniae and Haemophilus influenzae type b in children in the Gambia. Trop Med Int Health. (2000) 5(3):207–13. 10.1046/j.1365-3156.2000.00535.x 10747284

[4] Adegbola RA Falade AG Sam BE Aidoo M Baldeh I Hazlett D The etiology of pneumonia in malnourished and well-nourished gambian children. Pediatr Infect Dis J. (1994) 13(11):975–82. 10.1097/00006454-199411000-00008 7845751

[5] Klugman KP Madhi SA Huebner RE Kohberger R Mbelle N Pierce N. A trial of a 9-valent pneumococcal conjugate vaccine in children with and those without HIV infection. N Engl J Med. (2003) 349(14):1341–8. 10.1056/NEJMoa035060 14523142

[6] Madhi SA Klugman KP. A role for Streptococcus pneumoniae in virus-associated pneumonia. Nat Med. (2004) 10(8):811–3. 10.1038/nm1077 15247911 PMC7095883

[7] Eskola J Kilpi T Palmu A Jokinen J Haapakoski J Herva E Efficacy of a pneumococcal conjugate vaccine against acute otitis media. N Engl J Med. (2001) 344(6):403–9. 10.1056/nejm200102083440602 11172176

[8] Dagan R Givon-Lavi N Fraser D Lipsitch M Siber GR Kohberger R. Serum serotype-specific pneumococcal anticapsular immunoglobulin g concentrations after immunization with a 9-valent conjugate pneumococcal vaccine correlate with nasopharyngeal acquisition of pneumococcus. J Infect Dis. (2005) 192(3):367–76. 10.1086/431679 15995949

[9] Page MJ McKenzie JE Bossuyt PM Boutron I Hoffmann TC Mulrow CD The PRISMA 2020 statement: an updated guideline for reporting systematic reviews. Br Med J. (2021) 372:n71. 10.1136/bmj.n71 33782057 PMC8005924

[10] Sterne JAC Savović J Page MJ Elbers RG Blencowe NS Boutron I Rob 2: a revised tool for assessing risk of bias in randomised trials. Br Med J. (2019) 366:l4898. 10.1136/bmj.l4898 31462531

[11] Yan Q Li X Chen Y Li L Hu X. Efficacy of supportive care interventions for improving posttraumatic stress symptoms and resilience in family caregivers of cancer-affected children: a meta-analysis of randomized controlled trials. Worldviews Evid Based Nurs. (2025) 22(1):e12764. 10.1111/wvn.12764 39828279

[12] Bin Y Peng R Lee Y Lee Z Liu Y. Efficacy of Xuebijing injection on pulmonary ventilation improvement in acute pancreatitis: a systematic review and meta-analysis. Front Pharmacol. (2025) 16:1549419. 10.3389/fphar.2025.1549419 40308770 PMC12041077

[13] Nunes MC Cutland CL Klugman KP Madhi SA. Pneumococcal conjugate vaccine protection against coronavirus-associated pneumonia hospitalization in children living with and without HIV. mBio. (2021) 12(1):e02347-20. 10.1128/mBio.02347-20 33419872 PMC7845626

[14] Akinsola AK Ota MO Enwere GC Okoko BJ Zaman SM Saaka M Pneumococcal antibody concentrations and carriage of pneumococci more than 3 years after infant immunization with a pneumococcal conjugate vaccine. PLoS One. (2012) 7(2):e31050. 10.1371/journal.pone.0031050 22363544 PMC3282700

[15] Saaka M Okoko BJ Kohberger RC Jaffar S Enwere G Biney EE Immunogenicity and serotype-specific efficacy of a 9-valent pneumococcal conjugate vaccine (PCV-9) determined during an efficacy trial in the Gambia. Vaccine. (2008) 26(29–30):3719–26. 10.1016/j.vaccine.2008.04.066 18514974

[16] Mbelle N Huebner RE Wasas AD Kimura A Chang I Klugman KP. Immunogenicity and impact on nasopharyngeal carriage of a nonavalent pneumococcal conjugate vaccine. J Infect Dis. (1999) 180(4):1171–6. 10.1086/315009 10479145

[17] Huebner RE Mbelle N Forrest B Madore DV Klugman KP. Immunogenicity after one, two or three doses and impact on the antibody response to coadministered antigens of a nonavalent pneumococcal conjugate vaccine in infants of Soweto, South Africa. Pediatr Infect Dis J. (2002) 21(11):1004–7. 10.1097/00006454-200211000-00006 12442020

[18] Soininen A Nohynek H Lucero M Jousimies K Ugpo J Williams G Igg antibody concentrations after immunization with 11-valent mixed-carrier pneumococcal conjugate vaccine in efficacy trial against pneumonia among Filipino infants. Vaccine. (2009) 27(20):2680–8. 10.1016/j.vaccine.2009.02.059 19428879

[19] Cutts FT Zaman SM Enwere G Jaffar S Levine OS Okoko JB Efficacy of nine-valent pneumococcal conjugate vaccine against pneumonia and invasive pneumococcal disease in the Gambia: randomised, double-blind, placebo-controlled trial. Lancet. (2005) 365(9465):1139–46. 10.1016/s0140-6736(05)71876-6 15794968

[20] Lucero MG Nohynek H Williams G Tallo V Simões EA Lupisan S Efficacy of an 11-valent pneumococcal conjugate vaccine against radiologically confirmed pneumonia among children less than 2 years of age in the Philippines: a randomized, double-blind, placebo-controlled trial. Pediatr Infect Dis J. (2009) 28(6):455–62. 10.1097/INF.0b013e31819637af 19483514

[21] Durando P Faust SN Fletcher M Krizova P Torres A Welte T. Experience with pneumococcal polysaccharide conjugate vaccine (conjugated to CRM197 carrier protein) in children and adults. Clin Microbiol Infect. (2013) 19(Suppl 1):1–9. 10.1111/1469-0691.12320 24083785

[22] Ricketson LJ Bettinger JA Sadarangani M Halperin SA Kellner JD. Vaccine effectiveness of the 7-valent and 13-valent pneumococcal conjugate vaccines in Canada: an IMPACT study. Vaccine. (2022) 40(19):2733–40. 10.1016/j.vaccine.2022.03.048 35351324

[23] Sauerborn M van Beers MM Jiskoot W Kijanka GM Boon L Schellekens H Antibody response against betaferon® in immune tolerant mice: involvement of marginal zone B-cells and CD4+ T-cells and apparent lack of immunological memory. J Clin Immunol. (2013) 33(1):255–63. 10.1007/s10875-012-9783-z 22945588

[24] Tapiéro B Halperin SA Dionne M Meekison W Diaz-Mitoma F Zickler P Safety and immunogenicity of a hexavalent vaccine administered at 2, 4 and 6 months of age with or without a heptavalent pneumococcal conjugate vaccine: a randomized, open-label study. Pediatr Infect Dis J. (2013) 32(1):54–61. 10.1097/INF.0b013e3182717edf 23241989

[25] Smith DR. Herd immunity. Vet Clin North Am Food Anim Pract. (2019) 35(3):593–604. 10.1016/j.cvfa.2019.07.001 31590904

[26] Harboe ZB Dalby T Weinberger DM Benfield T Mølbak K Slotved HC Impact of 13-valent pneumococcal conjugate vaccination in invasive pneumococcal disease incidence and mortality. Clin Infect Dis. (2014) 59(8):1066–73. 10.1093/cid/ciu524 25034421

[27] Cohen C von Mollendorf C de Gouveia L Lengana S Meiring S Quan V Effectiveness of the 13-valent pneumococcal conjugate vaccine against invasive pneumococcal disease in South African children: a case-control study. Lancet Glob Health. (2017) 5(3):e359–69. 10.1016/s2214-109x(17)30043-8 28139443

[28] Shiri T Datta S Madan J Tsertsvadze A Royle P Keeling MJ Indirect effects of childhood pneumococcal conjugate vaccination on invasive pneumococcal disease: a systematic review and meta-analysis. Lancet Glob Health. (2017) 5(1):e51–9. 10.1016/s2214-109x(16)30306-0 27955789

[29] Tiley KS Ratcliffe H Voysey M Jefferies K Sinclair G Carr M Nasopharyngeal carriage of pneumococcus in children in England up to 10 years after 13-valent pneumococcal conjugate vaccine Introduction: persistence of serotypes 3 and 19A and emergence of 7C. J Infect Dis. (2023) 227(5):610–21. 10.1093/infdis/jiac376 36130327 PMC9978316

[30] Manoharan A Manchanda V Balasubramanian S Lalwani S Modak M Bai S Invasive pneumococcal disease in children aged younger than 5 years in India: a surveillance study. Lancet Infect Dis. (2017) 17(3):305–12. 10.1016/s1473-3099(16)30466-2 27956163

[31] Chen YN Hu YX Cao L Zheng H An ZJ. Analysis on the vaccination coverage of 13-valent pneumococcal conjugate vaccine in China from 2017 to 2021. Chin J Prevent Med. (2023) 57(10):1536–41. 10.3760/cma.j.cn112150-20221222-01221 37859368

[32] Hausdorff WP Feikin DR Klugman KP. Epidemiological differences among pneumococcal serotypes. Lancet Infect Dis. (2005) 5(2):83–93. 10.1016/S1473-3099(05)01280-6 15680778

[33] Blumental S Granger-Farbos A Moïsi JC Soullié B Leroy P Njanpop-Lafourcade BM Virulence factors of Streptococcus pneumoniae. Comparison between African and French invasive isolates and implication for future vaccines. PLoS One. (2015) 10(7):e0133885. 10.1371/journal.pone.0133885 26214695 PMC4516325

[34] Feikin DR Kagucia EW Loo JD Link-Gelles R Puhan MA Cherian T Serotype-specific changes in invasive pneumococcal disease after pneumococcal conjugate vaccine introduction: a pooled analysis of multiple surveillance sites. PLoS Med. (2013) 10(9):e1001517. 10.1371/journal.pmed.1001517 24086113 PMC3782411

[35] GBD 2019 Bacterial Pathogens Collaborators. Global mortality associated with 33 bacterial pathogens in 2019: a systematic analysis for the global burden of disease study 2019. Lancet. (2022) 400(10369):2221–48. 10.1016/s0140-6736(22)02185-7 36423648 PMC9763654

[36] Trück J Thompson A Morales-Aza B Clutterbuck EA Voysey M Clarke E Memory B cell response to a PCV-13 booster in 3.5year old children primed with either PCV-7 or PCV-13. Vaccine. (2017) 35(20):2701–8. 10.1016/j.vaccine.2017.03.079 28392142

[37] Mallet E Brachet E Fernsten P Laudat F Razmpour A Gruber WC. Immunogenicity and safety of CRM₁₉₇ conjugated 9-valent pneumococcal and meningococcal C combination vaccine in healthy infants. Vaccine. (2011) 29(34):5812–9. 10.1016/j.vaccine.2011.01.057 21296118

[38] Whitney CG Farley MM Hadler J Harrison LH Bennett NM Lynfield R Decline in invasive pneumococcal disease after the introduction of protein-polysaccharide conjugate vaccine. N Engl J Med. (2003) 348(18):1737–46. 10.1056/NEJMoa022823 12724479

[39] Ben-Shimol S Greenberg D Givon-Lavi N Schlesinger Y Somekh E Aviner S Early impact of sequential introduction of 7-valent and 13-valent pneumococcal conjugate vaccine on IPD in Israeli children <5 years: an active prospective nationwide surveillance. Vaccine. (2014) 32(27):3452–9. 10.1016/j.vaccine.2014.03.065 24690148

[40] Feemster K Buchwald UK Banniettis N Joyce JG Velentgas P Chapman TJ Immunogenicity of current and next-generation pneumococcal conjugate vaccines in children: current challenges and upcoming opportunities. Open Forum Infect Dis. (2024) 11(5):ofae220. 10.1093/ofid/ofae220 38770212 PMC11103622

[41] Scott JA Ojal J Ashton L Muhoro A Burbidge P Goldblatt D. Pneumococcal conjugate vaccine given shortly after birth stimulates effective antibody concentrations and primes immunological memory for sustained infant protection. Clin Infect Dis. (2011) 53(7):663–70. 10.1093/cid/cir444 21865175 PMC3166350

[42] Vardanjani HM Borna H Ahmadi A. Effectiveness of pneumococcal conjugate vaccination against invasive pneumococcal disease among children with and those without HIV infection: a systematic review and meta-analysis. BMC Infect Dis. (2019) 19(1):685. 10.1186/s12879-019-4325-4 31382917 PMC6683423

[43] Findlow H Borrow R. Interactions of conjugate vaccines and co-administered vaccines. Hum Vaccin Immunother. (2016) 12(1):226–30. 10.1080/21645515.2015.1091908 26619353 PMC4962715

[44] Janssens E Flamaing J Vandermeulen C Peetermans WE Desmet S De Munter P. The 20-valent pneumococcal conjugate vaccine (PCV20): expected added value. Acta Clin Belg. (2023) 78(1):78–86. 10.1080/17843286.2022.2039865 35171752

[45] Chen K Zhang X Shan W Zhao G Zhang T. Serotype distribution of Streptococcus pneumoniae and potential impact of pneumococcal conjugate vaccines in China: a systematic review and meta-analysis. Hum Vaccin Immunother. (2018) 14(6):1453–63. 10.1080/21645515.2018.1435224 29451838 PMC6037451

[46] Werren JP Troxler LJ Oyewole OR Ramette A Brugger SD Bruggmann R Carbon source-dependent changes of the structure of Streptococcus pneumoniae capsular polysaccharide with serotype 6F. Int J Mol Sci. (2021) 22(9):4580. 10.3390/ijms22094580 33925509 PMC8123889

[47] Karppinen S Toivonen L Schuez-Havupalo L Teros-Jaakkola T Waris M Auranen K Effectiveness of the ten-valent pneumococcal Haemophilus influenzae protein D conjugate vaccine (PHiD-CV10) against all respiratory tract infections in children under two years of age. Vaccine. (2019) 37(22):2935–41. 10.1016/j.vaccine.2019.04.026 31027929

[48] Carmona Martinez A Prymula R Miranda Valdivieso M Otero Reigada MDC Merino Arribas JM Brzostek J Immunogenicity and safety of 11- and 12-valent pneumococcal non-typeable Haemophilus influenzae protein D-conjugate vaccines (11vPHiD-CV, 12vPHiD-CV) in infants: results from a phase II, randomised, multicentre study. Vaccine. (2019) 37(1):176–86. 10.1016/j.vaccine.2018.07.023 30054160

