# Supplementary material for: Efficacy, immunogenicity, and safety of pneumococcal conjugate vaccine in children: a systematic review and meta-analysis
Source: Front Pediatr. 2025 Oct 1;13:1652946. doi: 10.3389/fped.2025.1652946 (PMC12520882; doi:10.3389/fped.2025.1652946)
Supplement: Supplementary file 2 [file Table2.docx]

**Table S2. Search strategy**

| ***PubMed***  "child*"[Title/Abstract] OR "adolescen*"[Title/Abstract] OR "infant*"[Title/Abstract] OR "juvenile"[Title/Abstract] OR "neonat*"[Title/Abstract] OR "newborn"[Title/Abstract] OR "teen*"[Title/Abstract] OR "youth*"[Title/Abstract] AND ("diplococcus pneumoniae"[Title/Abstract] AND "Pneumococcus"[Title/Abstract] AND "streptococcus pneumoniae"[Title/Abstract] AND "vaccin*"[Title/Abstract]) OR ("13"[All Fields] AND "vpnc"[Title/Abstract]) OR "13vpnc"[Title/Abstract] OR ("20"[All Fields] AND "vpnc"[Title/Abstract]) OR "20vpnc"[Title/Abstract] OR "apexxnar"[Title/Abstract] OR "moniarix"[Title/Abstract] OR "pcv 13"[Title/Abstract] OR "pcv13"[Title/Abstract] OR "phid cv"[Title/Abstract] OR ("pneu"[All Fields] AND "immune"[Title/Abstract]) OR ("PnuImune"[All Fields] AND "Vaccine"[Title/Abstract]) OR "ppv23"[Title/Abstract] OR "prevenar*"[Title/Abstract] OR "prevnar*"[Title/Abstract] OR "streptococcus pneumoniae vaccine"[Title/Abstract] OR "synflorix"[Title/Abstract] OR "v 110"[Title/Abstract] OR ("v"[All Fields] AND "114"[Title/Abstract]) OR "v110"[Title/Abstract] OR "v114"[Title/Abstract] OR "vaxneuvance"[Title/Abstract] AND "random*"[Title/Abstract] OR "RCT"[Title/Abstract] OR "randomized controlled trial"[Title/Abstract] OR "placebo"[Title/Abstract] AND (("diplococcus pneumoniae"[Title/Abstract] AND "Pneumococcus"[Title/Abstract] AND "streptococcus pneumoniae"[Title/Abstract] AND "vaccin*"[Title/Abstract]) OR ("13"[All Fields] AND "vpnc"[Title/Abstract]) OR "13vpnc"[Title/Abstract] OR ("20"[All Fields] AND "vpnc"[Title/Abstract]) OR "20vpnc"[Title/Abstract] OR "apexxnar"[Title/Abstract] OR "moniarix"[Title/Abstract] OR "pcv 13"[Title/Abstract] OR "pcv13"[Title/Abstract] OR "phid cv"[Title/Abstract] OR ("pneu"[All Fields] AND "immune"[Title/Abstract]) OR ("PnuImune"[All Fields] AND "Vaccine"[Title/Abstract]) OR "ppv23"[Title/Abstract] OR "prevenar*"[Title/Abstract] OR "prevnar*"[Title/Abstract] OR "streptococcus pneumoniae vaccine"[Title/Abstract] OR "synflorix"[Title/Abstract] OR "v 110"[Title/Abstract] OR ("v"[All Fields] AND "114"[Title/Abstract]) OR "v110"[Title/Abstract] OR "v114"[Title/Abstract] OR "vaxneuvance"[Title/Abstract]) AND ("random*"[Title/Abstract] OR "RCT"[Title/Abstract] OR "randomized controlled trial"[Title/Abstract] OR "placebo"[Title/Abstract]) |
| --- |
| ***Cochrane Library***  ((“Child”[mesh] OR “Infant”[mesh] OR “newborn”[mesh] OR “Adolescent”[mesh] OR Child* OR Adolescen* OR Infant* OR juvenile OR neonat* OR newborn OR Teen* OR Youth*) AND (“Streptococcus pneumoniae”[mesh] OR Diplococcus pneumoniae OR Pneumococcus OR Streptococcus pneumoniae OR “Vaccines”[mesh] OR vaccin*)) OR (13 vpnc OR 13vpnc OR 20 vpnc OR 20vpnc OR apexxnar OR moniarix OR pcv 13 OR pcv13 OR pf 06482077 OR pf06482077 OR phid cv OR pneu immune OR pneumo 23 OR pneumococcal vaccine* OR Pneumococcus vaccine OR pneumopur OR Pneumovax OR pnu immune OR pnu imune* OR PnuImune Vaccine OR ppv23 OR prevenar* OR prevnar* OR streptococcus pneumoniae vaccine OR streptopur OR streptorix OR synflorix OR v 110 OR v 114 OR v110 OR v114 OR vaxneuvance) AND (random* OR RCT OR randomized controlled trial OR placebo) |
| **Web of Science**  TS=(Child* OR Adolescen* OR Infant* OR juvenile OR neonat* OR newborn OR Teen* OR Youth*) AND TS=(random* OR RCT OR randomized controlled trial OR placebo) AND TS=(“Diplococcus pneumoniae” OR “Pneumococcus” OR “Streptococcus pneumoniae”) AND TS=vaccin*) OR TS=(“13 vpnc” OR “13vpnc” OR “20 vpnc “ OR “20vpnc” OR “apexxnar” OR “moniarix” OR “pcv 13” OR “pcv13” OR “pf 06482077” OR “pf06482077” OR “phid cv” OR “pneu immune” OR “pneumo 23” OR “pneumococcal vaccine*” OR “Pneumococcus vaccine” OR “pneumopur” OR “Pneumovax” OR “pnu immune” OR “pnu imune*” OR “PnuImune Vaccine” OR “ppv23” OR “prevenar*” OR “prevnar*” OR “streptococcus pneumoniae vaccine” OR “streptopur” OR “streptorix” OR “synflorix” OR “v 110” OR “v 114” OR “v110” OR “v114” OR “vaxneuvance”). |
| ***Embase***  ('streptococcus pneumoniae'/exp OR 'diplococcus pneumoniae':ab,ti OR pneumococcus:ab,ti OR 'streptococcus pneumoniae':ab,ti) AND ('vaccine'/exp OR vaccin*:ab,ti) OR ('pneumococcus vaccine'/exp OR '13 vpnc':ab,ti OR 13vpnc:ab,ti OR '20 vpnc':ab,ti OR 20vpnc:ab,ti OR apexxnar:ab,ti OR moniarix:ab,ti OR 'pcv 13':ab,ti OR pcv13:ab,ti OR 'pf 06482077':ab,ti OR pf06482077:ab,ti OR 'phid cv':ab,ti OR 'pneu immune':ab,ti OR 'pneumo 23':ab,ti OR 'pneumococcal vaccine*':ab,ti OR 'pneumococcus vaccine':ab,ti OR pneumopur:ab,ti OR 'pnu immune':ab,ti OR 'pnu imune*':ab,ti OR 'pnuimune vaccine':ab,ti OR ppv23:ab,ti OR prevenar*:ab,ti OR prevnar*:ab,ti OR 'streptococcus pneumoniae vaccine':ab,ti OR streptopur:ab,ti OR streptorix:ab,ti OR synflorix:ab,ti OR 'v 110':ab,ti OR 'v 114':ab,ti OR v110:ab,ti OR v114:ab,ti OR vaxneuvance:ab,ti) AND (random*:ab,ti OR rct:ab,ti OR 'randomized controlled trial':ab,ti OR placebo:ab,ti) AND ('child'/exp OR 'infant'/exp OR 'newborn'/exp OR 'adolescent'/exp OR 'juvenile'/exp OR child*:ab,ti OR adolescen*:ab,ti OR infant*:ab,ti OR juvenile:ab,ti OR neonat*:ab,ti OR newborn:ab,ti OR teen*:ab,ti OR youth*:ab,ti). |
